# Antibodies from multiple sclerosis patients preferentially recognize hyperglucosylated adhesin of non-typeable *Haemophilus influenzae*

**DOI:** 10.1038/srep39430

**Published:** 2016-12-23

**Authors:** Marthe T. C. Walvoort, Chiara Testa, Raya Eilam, Rina Aharoni, Francesca Nuti, Giada Rossi, Feliciana Real-Fernandez, Roberta Lanzillo, Vincenzo Brescia Morra, Francesco Lolli, Paolo Rovero, Barbara Imperiali, Anna Maria Papini

**Affiliations:** 1 Departments of Biology and Chemistry Massachusetts Institute of Technology 77 Massachusetts Ave., Cambridge, MA, USA; 2 French-Italian Interdepartmental Laboratory of Peptide and Protein Chemistry and Biology, University of Florence, 50019, Sesto Fiorentino, Italy; 3 Department of Neurosciences, Psychology, Drug Research and Child Health - Section of Pharmaceutical Sciences and Nutraceutics, University of Florence, Via Ugo Schiff 6, 50019, Sesto Fiorentino, Italy; 4 Department of Veterinary Resources, The Weizmann Institute of Science, Rehovot, 761001, Israel; 5 Department of Immunology, The Weizmann Institute of Science, Rehovot, 761001, Israel; 6 Department of Chemistry “Ugo Schiff”, University of Florence, Via della Lastruccia 13, 50019 Sesto Fiorentino, Italy; 7 Multiple Sclerosis Clinical Care and Research Centre, Department of Neurosciences, Reproductive Sciences and Odontostomatology, Federico II University, Via Sergio Pansini 5, 80131 Naples, Italy; 8 Department of Biomedical, Experimental and Clinical Sciences, University of Florence, Viale Morgagni 50, 50134 Firenze, Italy; 9 PeptLab@UCP and Laboratory of Chemical Biology EA4505, Université Paris-Seine, 5 Mail Gay-Lussac, 95031 Cergy-Pontoise, France

## Abstract

In autoimmune diseases, there have been proposals that exogenous “molecular triggers”, i.e., specific this should be ‘non-self antigens’ accompanying infectious agents, might disrupt control of the adaptive immune system resulting in serious pathologies. The etiology of the multiple sclerosis (MS) remains unclear. However, epidemiologic data suggest that exposure to infectious agents may be associated with increased MS risk and progression may be linked to exogenous, bacterially-derived, antigenic molecules, mimicking mammalian cell surface glycoconjugates triggering autoimmune responses. Previously, antibodies specific to a gluco-asparagine (N-Glc) glycopeptide, CSF114(N-Glc), were identified in sera of an MS patient subpopulation. Since the human glycoproteome repertoire lacks this uniquely modified amino acid, we turned our attention to bacteria, i.e., *Haemophilus influenzae*, expressing cell-surface adhesins including N-Glc, to establish a connection between *H. influenzae* infection and MS. We exploited the biosynthetic machinery from the opportunistic pathogen *H. influenzae* (and the homologous enzymes from *A. pleuropneumoniae*) to produce a unique set of defined glucosylated adhesin proteins. Interestingly we revealed that a hyperglucosylated protein domain, based on the cell-surface adhesin HMW1A, is preferentially recognized by antibodies from sera of an MS patient subpopulation. In conclusion the hyperglucosylated adhesin is the first example of an N-glucosylated native antigen that can be considered a relevant candidate for triggering pathogenic antibodies in MS.

Multiple sclerosis (MS) is the most common debilitating neurological disease of young adults in the developed world. It is a complex heterogeneous, inflammatory disorder characterized by a loss of the myelin sheath surrounding the nerve axons in the central nervous system (CNS). MS is considered an autoimmune disease in which the possible role of infections as triggering events is actively debated. In fact, it is unclear how adaptive immune responses are involved in CNS damage, and whether such responses are the pathogenic root cause or just an epiphenomenon in MS. The defined cause of MS is still unknown, but it is established that complex interactions between environmental factors and multiple gene products are involved^1^,^2^,^3^. Epidemiologic data have identified several infectious factors that may be associated with increased MS risk^4^. Emerging hypotheses consider that the progression of MS is linked to exogenous infectious agents expressing antigenic molecules, which mimic the structure and/or conformation of endogenous mammalian surface glycoproteins and/or glycolipids^5^,^6^. This molecular mimicry between self-molecules and exogenous antigens could lead to activation of autoreactive lymphocytes thereby establishing clinical symptoms. Although an antibody-mediated demyelination mechanism might contribute to the immunopathology of MS, this hypothesis has never been unequivocally demonstrated. In fact, the role of antibody recognition in MS is controversial and the cause of significant debate, possibly because antigens employed in immunoassays do not faithfully recapitulate the putative native entities implicated in triggering the immunologic response^7^,^8^,^9^.

Myelin Oligodendrocyte Glycoprotein (MOG), a glycoprotein in the CNS, has emerged as a potential autoantigen involved in demyelination processes fundamental to MS. While the importance of anti-MOG autoantibodies has been established in pediatric demyelination^10^, their role in adult MS patients is still speculative^11^,^12^. Intrigued by the possible role of MOG glycosylation in pathogenic autoantibody generation, a series of peptides and glycopeptides were investigated for their binding to autoantibodies present in MS patient sera^13^,^14^. This led to the identification of an antigenic probe termed CSF114(N-Glc) (Fig. 1a), which was used to detect, isolate, and characterize antibodies as potential biomarkers in a subpopulation of MS patients^15^,^16^. This was the first report that an aberrant asparagine-glucosylation (N-Glc) was implicated in triggering formation of autoantibodies in MS. The high specificity and affinity with which the CSF114(N-Glc) is recognized and binds to antibodies in MS patient sera were ascribed to two critical structural aspects of the biomarker: the N-linked β-d-glucopyranosyl moiety^17^ and a β-hairpin structure, which optimally exposes the minimal Asn(N-Glc) epitope, both essential for autoantibody recognition^14^. At the time that the CSF114(N-Glc) antigen was identified, there was a paucity of evidence for the presence of Asn(N-Glc) in eukaryotic proteins. Apart from a single report identifying an N-glucose moiety in the mammalian glycoprotein laminin^18^, this specific post-translational modification was virtually absent in eukaryotes, and observed only rarely in selected archaea^19^. We therefore concluded that it was unlikely that the antibodies that reacted with CSF114(N-Glc) had been elicited specifically to a human protein or a simple mutation to a human protein as is the case with neuromyelitis optica (NMO), which is an autoimmune disease with demyelination of the spinal cord and optic nerve with clinical similarity to MS but specifically marked by anti-human aquaporin-4 antibodies^20^,^21^. However, recent biochemical, analytical and bioinformatics approaches have uncovered an expansive repertoire of prokaryote-specific glycoprotein conjugates that by virtue of their cell-surface localization may represent putative important antigens involved in the development of autoimmune diseases^22^. Interestingly, in 2003 St Geme *et al*. first reported that protein asparagine-glucosylation is a modification found on HMW1 adhesin in non-typeable *Haemophilus influenzae*^23^,^24^. Subsequently, in 2008 it was established that the bacterial N-glucosylation is performed by the cytoplasmic HMW1C glycosyltransferase using UDP-glucose as the glycosyl donor (Fig. 1b). Interestingly, the structure of the homologous HMW1C from *Actinobacillus pleuropneumoniae* was reported in 2011^25^ and later, a detailed biochemical evaluation of the *Ap* HM1WC revealed a relaxed substrate specificity. Nevertheless, UDP-Glc is unequivocally the preferred substrate over UDP-Gal (UDP-Glc: *k*_cat_/*K*_M_ = 502.8 M^−1^ s^−1^, UDP-Gal: *k*_cat_/*K*_M_ = 1.3 M^−1^ s^−1^)^26^. The HMW1Cs from *A. pleuropneumoniae* and *H. influenzae* are some of the first examples of soluble bacterial protein glycosyltransferases that are capable of performing N-glycosylation with simple hexoses (i.e., glucose) on asparagine residues in conserved Asn-Xaa-Ser/Thr motifs^27^. We hypothesized that a bacterial infection, accompanied by cell-surface presentation of N-glucosylated adhesins, could stimulate autoreactive MS immune cells to trigger an antibody response through a molecular mimicry mechanism. Using the glycosylation machinery from *H. influenzae* (and homologues of *A. pleuropneumoniae*), we produced and purified glycoproteins with defined N-glucosylation patterns. These antigens were preferentially recognized by antibodies from MS patients. Moreover, the purified antibodies were shown to cross-react with the myelin in the spinal cord tissue in an experimental animal model of MS.

## Results

### Biochemical production of purified glucosylated adhesin fragments

**Figure 1:**
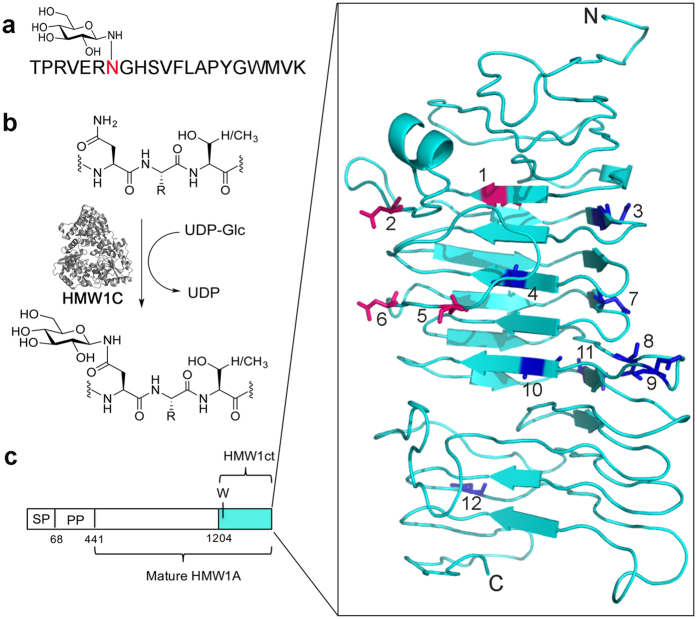
Schematic representation of the enzymatic reaction and putative N-glucosylation sites on HMW1ct. (**a**) Structure of CSF114(N-Glc). (**b**) Glucosylation of Asn in the consensus senquence N*X*(S/T), catalyzed by HMW1C, (PDB: 3Q3H). (**c**) Schematic representation of HMW1A, displaying the extended signal peptide (SP), pre-protein (PP), mature HMW1A, and the HMW1ct fragment (magnification is an I-TASSER^29^,^30^ model of HMW1ct, with glycosylation sites 1, 2, 5, 6 in magenta, and remaining sites in blue).

In order to identify the cross-reactivity between N-glucosylated adhesin fragments from *H. influenzae* and autoantibodies from MS patient sera, we established methods to generate a well-defined set of N-glucosylated protein antigens using biochemical techniques. We selected the C-terminal fragment of the HMW1A adhesin (residues 1205–1536, termed HMW1ct), which has been reported to be well expressed, soluble, and stably folded^28^. This domain of the HMW1A adhesin (see Supplementary Fig. 1 for the sequence) contains 12 putative N-glucosylation sites, of which some appear exposed on turns as predicted in the I-TASSER computational model of the protein (Fig. 1c)^29^,^30^. Mass spectrometry^24^ has revealed that sites 5, 6, and 7 (Fig. 1c) are found to be glycosylated in *H. influenzae*, but other reports suggest different glycosylation patterns both *in vitro* and *in vivo*^31^,^32^.

Similarly, the N-glucosyltransferase (HMW1C) homolog from *A. pleuropneumoniae* was employed for N-glucosylation because of its high expression and stability and the ability to produce glucosylated HMW1ct fragments^28^. The glucosylated HMW1ct antigen, **I**(**Glc**), was prepared by simultaneous co-expression of adhesin HMW1ct and N-glucosyltransferase HMW1C in *E. coli*^33^, that has an intracellular UDP-Glc concentration of 1–2 mM^34^. Using this method, **I**(**Glc**) was obtained as a mixture of three N-glucosylated HMW1ct variants containing 7, 8, and 9 glucose moieties on Asn in N*X*(S/T) sequons in a 1:1:1 ratio (Supplementary Table 1). Tryptic digest and MS/MS analysis was performed to determine which of the potential N-glycosylation sites were modified (Supplementary Table 2). Inspired by the model generated using the I-TASSER program, glycosylation sites in HMW1ct were sequentially removed using site-directed mutagenesis, giving antigens that display 5/6 N-Glc moieties, **II**(**Glc**), 4 spatially proximal N-Glc moieties (antigen **III**(**Glc**)), a set of four unrelated N-Glc positions (antigen **IV**(**Glc**)), and an antigen containing just two proximal N-Glc moieties (antigen **V**(**Glc**)) (Supplementary Table 1). These antigens were also produced in the corresponding non-glucosylated forms (**I**-**V**).

### Antibodies in MS patient sera recognize N-glucosylated adhesin HMW1ct-Glc antigens

**Figure 2:**
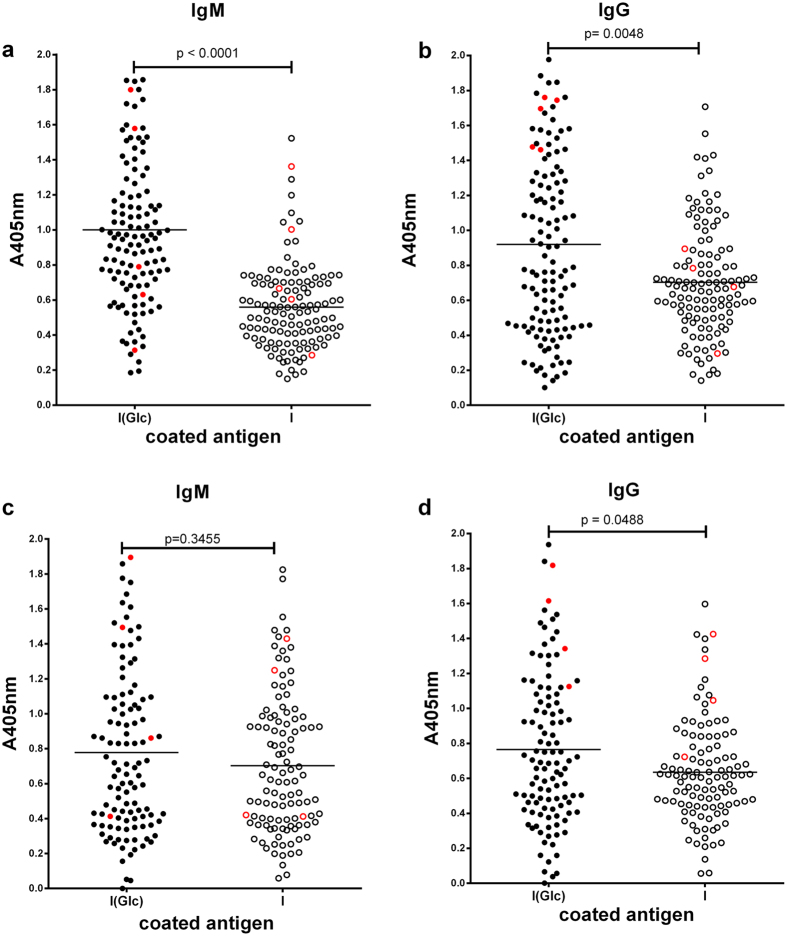
Data distribution of measured SP-ELISA absorbances. (**a**) Total IgM and (**b**) IgG titers in 126 MS patient sera detected by SP-ELISA, comparison between the antigen **I**(**Glc**), and the non-glucosylated analog **I**. (**c**) Total IgM and (**d**) IgG titers in 112 NBD sera detected by SP-ELISA, comparison between **I**(**Glc**) antigen and the non-glucosylated analog **I**. MS patient and NBD sera selected for the detailed analysis are marked as red dots and circles.

We investigated the ability of **I**(**Glc**) and the non-glucosylated protein **I** to detect IgM and IgG antibodies in sera of MS patients (Fig. 2a,b) and normal blood donors (NBDs) (Fig. 2c,d) using Solid-Phase ELISA (SP-ELISA).

The new hyperglucosylated protein **I**(**Glc**) detected IgM and IgG in MS patient sera and discriminated between the recruited population of 126 MS patients and 112 NBDs (IgM: p < 0.0003, IgG: p < 0.0141; Supplementary Fig. 2). Interestingly, the synthetic mono-glucosylated antigenic probe CSF114(N-Glc) was previously reported to discriminate between patients and NBDs with a similar trend, but much lower significance in the case of IgG (Supplementary Fig. 3)^14^,^15^, suggesting that hyperglucosylation of the bacterial adhesin protein, leading to the antigen **I**(**Glc**), favorably affects antibody recognition in MS. At variance, the non-glucosylated protein **I** did not discriminate between MS and NBD sera, and NBD sera presented a similar level of antibodies interacting with both the glucosylated **I**(**Glc**) and non-glucosylated **I** antigens. The relatively high titers observed for both antigens **I** and **I**(**Glc**) with NBD sera is not surprising as *H. influenzae* is a rather ubiquitous human pathogen to which most adults have been exposed. This results in the development of antibodies against epitopes that do not include the Glc moiety, but are shared by both **I** and **I**(**Glc**) (Supplementary Fig. 2). Significantly, the MS patient sera revealed to be highly populated with antibodies against the hyperglucosylated protein **I**(**Glc**), which suggests the recognition of an epitope specifically displayed on **I**(**Glc**).

### Antibody detection in MS sera is N-Glc dependent

The relatively high antibody titers obtained from the NBD sera (Fig. 2c,d) suggest that common protein epitopes shared by antigens **I**(**Glc**) and **I** may interfere with the detection of significant and distinct epitopes introduced by protein N-glucosylation. To quantify the antibody binding to **I**(**Glc**) and non-glucosylated analog **I** in both MS patient and NBD sera, an inhibition experiment was performed. A significant difference in antibody binding between glucosylated antigen **I**(**Glc**) (pIC_50_ = 7.67 ± 0.28–8.65 ± 0.10) and non-glucosylated antigen **I** (pIC_50_ = <5.0) was observed for MS patient sera (Table 1). As anticipated, based on the total antibody titer experiments (Fig. 2), little difference in antibody binding was observed between antigens **I**(**Glc**) (pIC_50_ = 6.37 ± 0.52–7.20 ± 0.29) and **I** (pIC_50_ = 5.92 ± 0.52–7.84 ± 0.42) in NBD sera (Table 1). This observation holds true independently from CSF114(N-Glc) positivity. In fact, two of the NBD samples are CSF114(N-Glc) positive (NBD2 and NBD4), while two are negative (NBD1 and NBD3).

**Figure 3:**
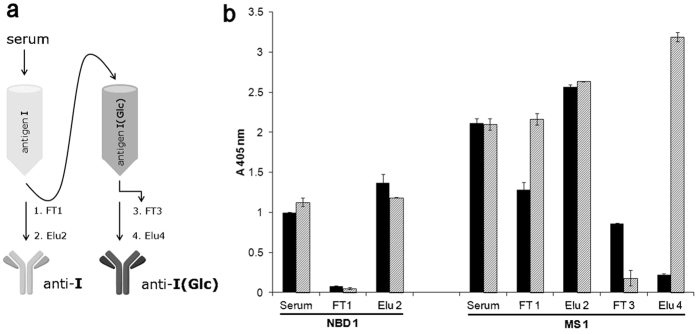
Immunoaffinity purification of antibodies from one representative NBD and one MS serum. (**a**) Schematic process of antibody fractionation. (**b**) SP-ELISA of antibody fractions obtained from two sequential sepharose columns bearing the immobilized non-glucosylated **I** and hyperglucosylated **I**(**Glc**). Antigen **I** designated as solid bars and antigen **I**(**Glc**) designated as gray bars. In the case of NBD serum, flow through 1 (**FT1**) and eluted fraction 2 (**Elu2**) were collected. In the case of MS serum, flow through 1 (**FT1**), eluted fraction 2 (**Elu2**), flow through 3 (**FT3**), eluted fraction 4 (**Elu4**) were collected. Each point is mean ± s.d. for n = 3 independent experiments. The ELISA absorbance in serum can be explained considering that IgG concentration in the whole sera (10 mg/ml) is 300 times higher than in eluted fractions (0.03 mg/ml).

Next, we explored the hypothesis that the N-Glc modification is a key determinant only for MS serum antibodies and that antibodies in NBD sera recognize protein epitopes shared by both the modified and unmodified proteins. Using the purified antigens **I**(**Glc**) and **I**, an immunoaffinity-based fractionation was performed for representative MS (MS 1) and NBD (NBD 1) sera, as outlined schematically in Fig. 3a.

The NBD1 and the MS1 sera were applied separately to a Sepharose column bearing immobilized non-glucosylated adhesin **I** and the flow-through (FT1) and elution (Elu2) were collected. In the case of MS1, the un-retained FT1 fraction was loaded on a second column, bearing the immobilized glucosylated protein antigen **I**(**Glc**). Retained IgGs were eluted from both columns separately (Elu2 and Elu4), and their activities were determined by SP-ELISA (Fig. 3b).

The FT1 from NBD1 displayed a remarkable decrease in IgG antibody recognition both to **I**(**Glc**) and the non-glucosylated **I**, suggesting that all antibody reactivity had been retained on the **I**-immobilized column, and that these antibodies have a similar titer for both antigens (Fig. 3b). This confirms that antibodies in NBD sera recognize structural protein epitopes rather than those associated with N-glucosylation (Supplementary Fig. 4). In contrast, with the MS1 serum, FT1 only displayed a significant decrease in anti-**I** antibodies, which had been retained on the first column (Fig. 3b). Indeed, when the resulting elution (Elu2) was purified with **I**(**Glc**)-immobilized Sepharose (second column), a high antibody reactivity with **I**(**Glc**) resulted (Elu4, Fig. 3b), which was not concentration dependent (Supplementary Fig. 5). These results together strongly support that MS patient sera in contrast to the NBD sera have a relatively high level of antibodies against the hyperglucosylated antigen **I**(**Glc**).

### MS antibodies detect an epitope shared by CSF114(N-Glc) and HMW1ct-Glc

We then set out to investigate the cross-reactivity between the N-glucosylated adhesin antigen **HMW1ct-Glc** and anti-CSF114(N-Glc) IgG antibodies by competitive ELISA^35^. The possible cross-reaction between anti-CSF114(N-Glc) antibodies in MS patient sera and the N-glucosylated antigens (**I**(**Glc**)**-V**(**Glc**)) was tested in a selected population of representative MS patient and NBD sera (Table 2). Results clearly showed that the hyper N-glucosylated **I**(**Glc**) was able to inhibit the binding of anti-CSF114(N-Glc) IgG antibodies in sera from a selected population of representative MS patients (mean pIC_50_ = 9.07 ± 0.42). In contrast, non-glucosylated protein **I** did not inhibit, or only inhibited MS serum binding at significantly higher concentrations (mean pIC_50_ = <6.0), confirming the fundamental role of the N-glucosyl moieties on the adhesin protein for the interaction with anti-N-glucosyl antibodies in MS sera (Table 2, entries 1 and 6, respectively). In order to more specifically define the N-Glc epitope(s) involved in antibody recognition, the HMW1ct mutants (**II**(**Glc**)**-V**(**Glc**) and **II-V**) were also tested for their binding to anti-CSF114(N-Glc) IgG antibodies. Interestingly, antigens **II**(**Glc**)-**IV**(**Glc**), containing 5/6 and 4 differentially-spaced N-Glc moieties, displayed a similar binding affinity to the hyper-N-glucosylated antigen **I**(**Glc**) (Table 2, entries 2–4 and 1, respectively), with negligible binding of the non-glucosylated counterparts **II**-**IV** (Table 2, entries 7–9). Antigen **V**(**Glc**) however, displaying only two N-Glc moieties, showed a significant reduction in binding affinity to anti-CSF114(N-Glc) IgG antibodies (mean pIC_50_ = 7.19 ± 0.40), to an extent comparable to the binding affinity of CSF114(N-Glc) itself (Table 2, entries 5 and 11, respectively). As the difference in affinity between antigens **I**(**Glc**) and **V**(**Glc**) is almost 2 log units (Table 2, entries 1 and 5, respectively), this is a very significant decrease, even taking into account the different number of N-Glc epitopes in the various samples (7/8/9 versus 2, respectively). Convincingly, a similar trend in binding of antigens **I**-**V** and **I**(**Glc**)-**V**(**Glc**) with anti-**I**(**Glc**) antibodies was obtained in a similar inhibition experiment (Supplementary Fig. 6). Moreover, Surface Plasmon Resonance (SPR) studies revealed a similar binding affinity of **I**(**Glc**) and CSF114(N-Glc) for purified anti-**I**(**Glc**) IgG antibodies, confirming their cross-reactivity (Supplementary Fig. 7). These results strongly indicate that both the number and the specific presentation of the N-glucosyl moieties play an important role in antibody recognition in MS patient sera.

### Anti-I(Glc) antibodies bind to myelin in the CNS

**Figure 4:**
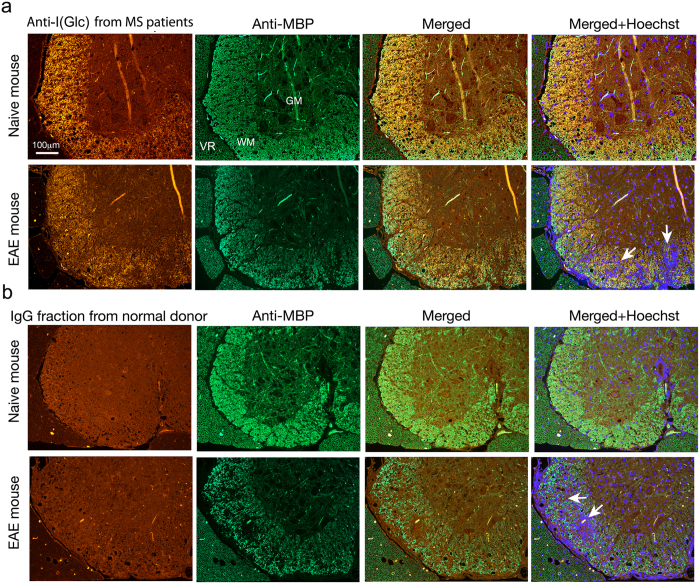
Immunofluorescent staining of mouse spinal cord sections by anti-I(Glc) antibodies. Coronal lumbar spinal cord sections from naïve mice as well as from mice inflicted with experimental autoimmune encephalomyelitis (EAE, grade 4, three weeks after disease induction), were incubated with: (**a**) anti-**I**(**Glc**) antibodies from a representative MS patient serum, (**b**) Total IgG fraction from a normal donor (NBD). Binding to the spinal cord tissue was indicated by fluorescent labeling with anti-human antibody (red). Note that the positive staining of anti-**I**(**Glc**) antibodies overlaps the myelin depicted by staining with antibody to myelin basic protein (MBP, green). Staining by anti-**I**(**Glc**) antibodies is lost in the spinal-cord white matter (**WM**) of EAE-mice, at sites of inflammation (depicted by Hoechst nuclear staining of the infiltrating cells, blue), in parallel to the demyelination. Anti**-I**(**Glc**) antibody binding is restricted to the spinal cord WM while no staining is observed in the gray matter (**GM**) or in the periphery ventral roots (**VR**). Arrows depict inflammatory sites in sections of EAE mice. The linear structures stained at the center of gray matter (**a**) are non-relevant staining resulting from folds in the tissue.

**Figure 5:**
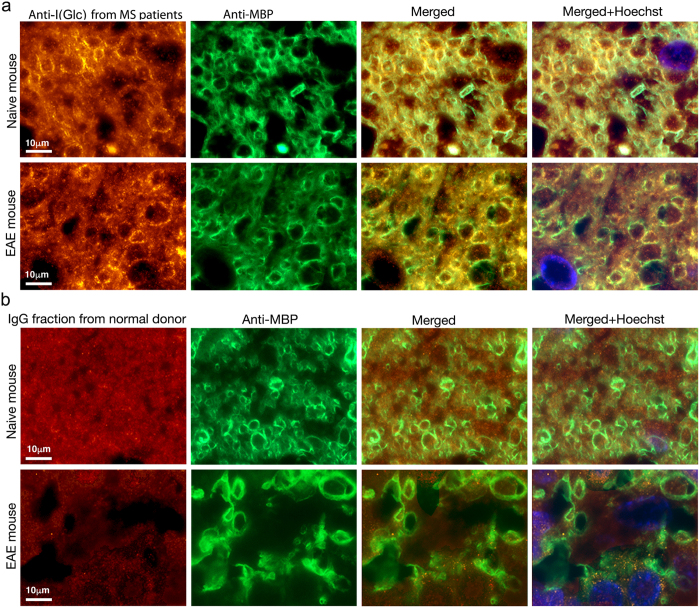
A higher magnification of the white matter taken from the different sections in Fig. 4, showing in further detail the immunofluorescent staining by anti-I(Glc) antibodies. Coronal lumbar spinal cord sections from naïve mice as well as from mice inflicted with experimental autoimmune encephalomyelitis (EAE, grade 4, three weeks after disease induction), were incubated with: (**a**) anti-**I(Glc)** antibodies from a representative MS patient serum, (**b**) Total IgG fraction from a normal donor (NBD). Binding to the spinal cord tissue was indicated by fluorescent labeling with anti-human antibody (red). Note that the positive staining of anti-**I(Glc)** antibodies overlaps with the myelin depicted by staining with antibody to myelin basic protein (MBP, green). Staining by anti-**I(Glc)** antibodies is lost in spinal cords of EAE-mice, at sites of inflammation (depicted by Hoechst nuclear staining of the infiltrating cells, blue), in parallel to the demyelination.

To demonstrate the biological significance of the binding of antibodies from MS patient sera to hyperglucosylated antigen **I**(**Glc**), immunohistochemistry experiments on normal and experimental autoimmune encephalomyelitis (EAE) mouse spinal cords were performed. In this experiment, the cross-species ability of purified anti-hyperglucosylated adhesin IgG antibodies from sera of a representative MS patient to recognize CNS tissue was compared to the binding of the total IgG fraction from NBD sera. The CNS myelin of mice is an accepted system for screening pathogenically relevant and immunoreactive antigens because these antigens are well conserved relative to the human counterparts. Moreover, the murine tissues are available for rapid processing thereby avoiding problems with protein degradation as is often encountered in human brain autopsy samples. Additionally, EAE is a relevant and broadly accepted model for MS and considerable insight into MS pathophysiology has been achieved using this model^4^. Anti-hyperglucosylated adhesin antibodies were incubated with spinal cord sections of naïve healthy mice and of mice inflicted with EAE, harvested three weeks after disease induction, when hind and forelegs were paralyzed (grade 4)^36^. In parallel, spinal cord sections of the same mice were incubated with the total IgG fraction from the normal blood donor sera. It should be noted that purified anti-**I**(**Glc**) antibodies from the representative MS1 patient serum were used in immunohistochemistry experiments (Figs 4a and 5a), while the total IgG fraction from an NBD representative serum (NBD1) was used in the control experiment depicted in Figs 4b and 5b because the titer of anti-**I**(**Glc**) antibodies, purified by immunoaffinity chromatography, is too low in NBD serum. The anti-**I**(**Glc**) antibodies positively stained the mouse spinal cord (Figs 4a and 5a), whereas no staining was observed in the control experiment with IgGs from NBD (Figs 4b and 5b). Interestingly, while it cannot be excluded that negative staining with IgGs from NBD might be simply due to insufficient titer to anti-**I**(**Glc**) antibodies, staining by anti-**I**(**Glc**) antibodies from MS1 was restricted to the white matter region, overlapping the myelin (depicted by staining with antibodies to myelin basic protein, MBP). Intense anti-**I**(**Glc**) staining was particularly manifested in the spinal cord white matter of naïve mice, whereas in EAE-mice, at sites of inflammation (depicted by Hoechst nuclear staining of the infiltrating cells), staining by anti-**I**(**Glc**) antibodies was lost, in parallel to the demyelination. Anti-**I**(**Glc**) antibodies did not bind to the spinal cord neurons located in the grey matter, or to the myelin in the peripheral nervous system. These findings indicate that anti-glucosylated adhesin antibodies from MS patients can bind to the myelin in the mouse CNS. The observation that after demyelination in the EAE model little myelin remains for recognition by anti-**I(Glc)** antibodies further supports the hypothesis that these antibodies recognize myelin.

## Discussion

Genome sequencing, in combination with biochemical, bioanalytical, and bioinformatic approaches^37^,^38^,^39^,^40^ has uncovered the diverse glycoconjugate repertoire of many microbial pathogens and revealed a prevalence of prokaryote-specific saccharides and glycosidic linkages in the conjugated glycan structures decorating bacterial cell surfaces^41^,^42^,^43^,^44^. The abundance of these non-human glycoconjugates on the cell surfaces of infectious microorganisms makes them potential molecular candidates for promoting immune system evasion by molecular mimicry of host cell-surface glycans^45^. The same molecular mimicry that affords the pathogen avoidance from immune surveillance however, may present serious additional consequences to the host organism stemming from the same host glycans being recognized as foreign thus activating an autoimmune response. One of the best understood examples of a microbial infection leading to a post infection autoimmune disease comes from studies on the Gram-negative bacterium *Campylobacter jejuni*, which is the leading cause of gastroenteritis in humans^46^. While the actual *C. jejuni* infection symptoms clear in a few days, epidemiological studies show a significant correlation between *C. jejuni* infections and Guillian-Barré syndrome (GBS), which manifests in acute neuromuscular paralysis and approximately 25% of GBS patients have developed symptoms after infection with *C. jejuni*^47^. For GBS there is now compelling evidence, including a significant and disease-correlated increase in anti-GM1 IgG titers, that molecular mimicry of the host GM1 ganglioside by the *C. jejuni* lipooligosaccharide ultimately results in the autoimmune neuropathy condition^48^. Moreover, Sakiyama *et al*. recently reported 4 cases of encephalomyelitis correlated to an unknown pathogen. On the basis of the genomic studies and the ultrastructural analysis of the pathogen, they proposed a new disease entity having archaeal features^49^.

The current study starts from the critical observation that antibody titers against a glycopeptide designated as CSF114(N-Glc) are increased in the sera of MS patients^14^. In a longitudinal study of untreated relapsing-remitting multiple sclerosis (RR-MS) patients for up to 6 months, it was demonstrated that levels of anti-CSF114(N-Glc) antibodies paralleled intensity of clinical activity and number of brain lesions positive to Gd-enhanced Magnetic Resonance Imaging^14^. Despite many efforts to clarify the specificity or functional significance of antibodies in MS, their origin and role remain elusive. Therefore, the possibility of characterizing antibodies cross-reacting with bacterial antigens, might contribute to the understanding of immunopathology^50^.

With these considerations in mind, we turned our attention to the bacterial glycoconjugates of human pathogens. N-Glc is a prokaryote-specific modification that is found in selected Gram-negative bacteria, where it is most commonly found on cell-surface proteins such as (autotransporter) adhesins – biosynthesized as part of the three-protein HMW cluster including the N-glucosyl tranfserase HMW1C. These adhesins are abundantly displayed on the bacterial cell surface, and because they are often extensively glycosylated, the glycan structures are likely to be presented in a multivalent manner, which would potentially favor the emergence of a robust immunologic response.

We present evidence of a strong connection between MS and the pathogen *Haemophilus influenzae*, which is a common cause of upper respiratory infection in humans. The biochemical construction of N-glucosylated protein antigens using the biosynthetic machinery from *H. influenzae* and *A. pleuropneumoniae* was critical to isolate purified antibodies from MS patient sera. Evidently, introduction of at least four N-glucosyl moieties on the *H. influenzae* C-terminal adhesin fragment HMW1ct(1205–1526) is essential for the identification of the highest affinity antibodies in multiple sclerosis. The purified MS antibodies were used in SP-ELISA titrations, inhibition, and SPR experiments, to demonstrate specific recognition of the N-Glc epitope. Finally, in immunohistochemistry experiments, the purified MS antibodies showed intense and specific staining to the myelin in the spinal cord white matter, in contrast to the absence of staining by total IgG antibodies from normal blood donor.

We conclude that the *H. influenzae* hyperglucosylated adhesin **I**(**Glc**) is the first example of an N-glucosylated antigen that can be considered a relevant candidate for triggering pathogenic antibodies in multiple sclerosis. Our data are in agreement with a recent report on microbial infections and degenerative neurodiseases^51^ supporting that microbes can cause chronic as well as acute diseases. Some microbes can remain latent in the body with the potential for reactivation, the effects of which might occur years after initial infection. People can be infected but not necessarily affected, such that ‘controls’, even if infected, are asymptomatic.

With these new findings, the foundation is established for determining the nature of the molecular mimicry mechanism, and for elucidating the human protein target(s), which are cryptic mimics recognized by anti-hyperglycosylated adhesin antibodies in Multiple Sclerosis.

## Methods

### Patient criteria and descriptions

Multiple Sclerosis (MS) patient sera samples were collected in two Multiple Sclerosis Centers: The Multiple Sclerosis Clinical Care and Research Centre, Department of Neurosciences, Reproductive Sciences and Odontostomatology, Federico II University (Naples, Italy) and the Azienda Ospedaliera Universitaria Careggi, Clinica Neurologica, University of Florence (Florence, Italy). Normal Blood Donor (NBD) patient sera samples were collected by F. Lolli at the Azienda Ospedaliera Universitaria Careggi, Immunotransfusion Centre, University of Florence (Florence, Italy).

MS patient sera samples were collected from 126 patients (42 males) aged from 19 to 65 years (mean age 39 years). The patients fulfilled established international diagnostic criteria^52^. Total IgM and IgG titers were measured by SP-ELISA in all the MS patient sera samples. Five relapsing-remitting MS patients (MS1-MS5, age 35 to 58 years; 3 males) were subsequently selected for detailed analysis among patients displaying IgG > 1 to CSF114(N-Glc) and IgG > 1.5 to **I**(**Glc**). MS1, displaying high IgG titer also to I (>1), was selected for immunoaffinity purification of anti-**I**(**Glc**) antibodies used for immunohistochemistry and Surface Plasmon Resonance experiments. The age of disease onset ranged from 10 to 40 years. The clinical findings, the EDSS and the clinical course of the MS patients were evaluated at the time of blood sampling and follow-up. MRI examinations were made for diagnostic purposes with typical MS findings. In seropositive patients, disease duration was between 6 and 9 years, the EDSS score was between 2.5 and 4. Four of these patients (MS2-MS5) were in treatment with interferon-beta and MS1 was an untreated patient.

As controls, we collected 112 Normal Blood Donor (NBD) sera samples matched for age and sex with MS patient sera samples. Total IgM and IgG titers were measured by SP-ELISA using all the NBD sera samples as controls. Four NBD sera samples (NBD1-NBD4) were subsequently selected for detailed analysis among those displaying IgG > 1 to **I**(**Glc**) and **I**; two out of these four were IgG negative to CSF114(N-Glc) (NBD1 and NBD3), while two were positive (NBD2 and NBD4). NBD1 was selected as control in immunoaffinity purification experiments.

The present study was conducted in accordance with the Declaration of Helsinki. All experimental protocols performed were approved by the Ethics Committee of the University Hospitals above mentioned involved in this study. Written informed consent forms were obtained from all MS patients and NBD subjects for sampling and research. All the methods were carried out in accordance with relevant approved guidelines and regulations.

### Cloning

The gene encoding the HMW1ct corresponding to amino acids 1205–1536 was synthesized commercially after gene optimization for *E. coli*, and obtained in the pUC57 vector (Genscript). Using *KpnI* and *SacI* restriction sites, the gene was amplified by PCR and ligated into the pET-45b (+) vector encoding carbenicillin resistance (Novagen). A tryptophan residue was introduced in the N-terminal overhang by transforming Gly to Trp using QuickChange mutagenesis. HMW1ct mutants were designed by either substituting the Asn residue for Glu (N → Q) or substituting the Ser/Thr residue for Ala (S/T → A). The resulting mutants were constructed using the Quik Change II site-directed mutagenesis kit and the mutagenic primers sets (Supplementary Table 3) according to the manufacturer’s instructions. The gene encoding the HMW1C-like glucosyltransferase was obtained from *Actinobacillus pleuropneumoniae* genomic DNA (ATCC: 27088D-5) and inserted into the pET-24a (+) vector (Novagen), as described previously^33^,^26^. The vector-encoded C-terminal His_6_-tag was removed by introducing a stop codon after the *hm1C* gene using the primers in Supplementary Table 4 in a PCR protocol, and reintroducing the amplified gene in the pET-24a (+) to retain kanamycin resistance. The plasmids used in this study are described in Supplementary Table 5.

### General protocol for the preparation of N-glucosylated HMW1ct proteins via co-expression of HMW1ct and HMW1C

Transformation of the two plasmids pET45::*His*_*6*_*-hmw1ct* and pET24::*hmw1C-stop* was initiated by mixing both plasmids (0.5 μL) into BL21 cells (50 μL). After heat shock, the cells were diluted with SOC media (200 μL) and incubated at 37 °C for 90 min. A 100 μL aliquot was plated out on carbenicillin/kanamycin/chloramphenicol plates and incubated at 37 °C overnight. Colonies were selected for growing in a 5-mL overnight culture. Subsequently, the bacteria were grown to an OD_600_ of 0.6–0.8 at 37 °C, and expression was induced with 1 mM IPTG, followed by incubation at 16 °C overnight. Subsequently, bacterial cells were harvested, resuspended in lysis buffer (50 mM HEPES pH 7.5, 100 mM NaCl, 10% glycerol) and disrupted using sonication (3 × 1 min at 40% amplitude) in the presence of protease inhibitor (10 μL per gram of cells). After centrifugation at 35,000 × g for 65 min at 4 °C, the clear supernatant was applied to a suspension of Ni^2+^-NTA beads at 4 °C for 1 h. The beads were transferred to a frit-containing column, the flow-through was collected, and the beads were subsequently washed with washing buffer (30 mM imidazole, 50 mM HEPES pH 7.5, 300 mM NaCl, 5% glycerol) and both glucosylated HMW1ct and HMW1C were eluted with elution buffer (300 mM imidazole, 50 mM HEPES pH 7.5, 300 mM NaCl, 5% glycerol). Protein fractions were pooled and dialyzed into 20 mM Tris pH 8.0 and 20 mM NaCl. The protein mixtures were loaded onto a 5-mL anion exchange column (HiTrap Q FF) and eluted with a linear gradient of 20 mM Nacl-1M NaCl using an AKTA FPLC system, to facilitate separation of glucosylated HMW1ct and HMW1C. The fractions containing **I**(**Glc**) were pooled, concentrated, and dialyzed (50 mM HEPES pH 7.5, 150 mM NaCl, 5% glycerol) to give a total yield of ~16 mg of **I**(**Glc**) from a 1 L culture.

### *In vitro* glucosylation of V(Glc)

Using the co-expression protocol, over-glucosylation of antigen **V** was observed. Instead of glycosylation of the two remaining sites, a mixture of proteins with 2, 3, and 4 glucose units was observed in a ratio of 2:2:1 respectively. Glucosylation of non-canonical sites has been reported previously^26^ and it was expected to be a result of the high expression levels of HMW1C. Using an *in vitro* glucosylation protocol, the glucosylation level of antigen **V** was greatly reduced, resulting in pure twice glycosylated antigen **V**(**Glc**).

After optimization studies, in which the concentration of **V** and equivalents of HMW1C were varied, optimal conditions were obtained to produce **V**(**Glc**) with only two Glc moieties: 50 μM **V**, 500 nM HMW1C, 500 μM UDP-Glc in a final volume of 10 mL. The reaction was incubated at RT overnight, and subsequently purified using Ni^2+^-NTA.

### Tryptic digest and MS/MS analysis of I(Glc)

A sample containing 4.6 μg of the purified **I**(**Glc**) was diluted with 100 μL 8 M urea (in 50 mM ammonium bicarbonate pH 8.5). The sample was heated at 55 °C for 1.5 h, after which time 12 μL 300 mM iodoacetamide in ammonium bicarbonate was added. The mixture was incubated at RT in the dark for 60 min, and subsequently transferred to a 10,000 MWCO spin filter. A 300 μL aliquot of ammonium carbonate was added, and the mixture was centrifuged at 14,000 × *g* to reduce the volume in the filter to below 50 μL. This was repeated with 300 μL and 400 μL ammonium carbonate, followed by transfer of the residue to a clean eppendorf tube. Then 6 μL of a freshly prepared trypsin solution (1 μg per 30 μL 50 mM ammonium carbonate) was added to the sample, and the mixture was incubated at RT overnight. Subsequently, 5.6 μL 10% formic acid in water was added to acidify the mixture, and it was analyzed using LC-MS/MS (Supplementary Table 2).

### Measurement of antibody affinity by competitive ELISA and statistical analysis

The experimental strategy involves immobilization of hyperglucosylated antigen **I**(**Glc**), followed by incubation with a serum in the presence of a concentration range of the competing species (in this case, antigens **I**(**Glc**) or **I**). The resultant antibody binding to the immobilized antigen is quantified using a fluorescent secondary antibody, and the absorbance units across the concentration range can be converted to a pIC_50_ value (Table 1). The relative mean affinity of antibodies was measured by following the method published by Rath *et al*.^35^. The semi-saturating dilution was calculated from the preliminary titration curves (absorbance, 0.7). At this dilution, antibodies were pre-incubated with increasing antigen concentrations (1 h at 25 °C). Bound antibodies were revealed by SP-ELISA, and the relative mean affinity of those antibodies for the different inhibitors was expressed by pIC_50_ values calculated through non-linear regression curve fit using GraphPad Prism 6.0.

### Sequential immunoaffinity chromatography

**I**(**Glc**) (3 mg) and the non-glucosylated adhesin **I** (3 mg) were dissolved in NaHCO_3_ (0.1 M, 3 mL) containing 0.5 M NaCl, pH 8.3, and were coupled to CNBr-Sepharose (200 mg) matrix (Sigma). The NBD1 and the MS1 sera (3 mL) positive to both CSF114(N-Glc) and **I**(**Glc**) (diluted 1:10 in PBS) were passed three times through the non-glucosylated adhesin **I** column pre-equilibrated with PBS, pH 7.2. In the case of MS1, the un-retained flow-through fraction was directly loaded onto the column and recirculated three times. Adsorbed antibodies were eluted using glycine (0.1 M, 10 mL, pH 2.6) and recovered in D-PBS buffer pH 7.2. Protein content of specific anti-**I**(**Glc**) purified IgG antibodies was determined to be 46.5 μg/ml by UV spectroscopy measuring the absorbance of the solution at 280 nm (Shimadzu UV-1601PC) considering that the extinction coefficient is 1.4 for a solution of 1 mg/mL. The efficiency of this sequential immunoadsorbtion was confirmed by SP-ELISA.

The total IgG fraction from NBD1 serum was obtained by Protein G affinity chromatography using a prepacked column (Amersham Biosciences, NJ) according to manufacturer’s instructions. Protein content of the total IgG fraction from NBD1 serum was determined to be 1.7 mg/mL using the method above described.

### SP-ELISA for antibody titer determination

Specific anti-**I**(**Glc**) IgG and anti-**I** antibodies were evaluated in SP-ELISA following a previously validated protocol^14^. Although the use of SP-ELISA may result in diminished specificity and statistical significance of antibody recognition due to the possibility of multiple non-relevant epitopes in the protein, it allows screening of a large number of patient sera in a relatively short time using minimal amounts of surface-coated antigen.

### Biosensor Analysis

All experiments were carried out on a Biacore T100 (GE Healthcare, Sweden). All solutions and buffers were prepared with milliQ water obtained by a Sartorius system (arium 611 VF). Sensor chips CM5 (carboxymethylated dextran), amine coupling kit (EDC/NHS (50:50), and Running Buffer HBS-EP+ 10× (0.1 mol/L HEPES, 1.5 mol/L NaCl, 30 mmol/L EDTA, 0.5% v/v p20) were purchased by GE Healthcare. Running Buffer was diluted ten times with milliQ water at pH 7.4. Sodium acetate and sodium hydroxide (Carlo Erba, Milano). Each antigen was immobilized on a different channel of the chip CM5-type according to the amine coupling strategy using a concentration of 10 μg/mL in immobilization buffer at 10 μL/min for 420 s and 60 s. Unreacted groups on the sensor chip surface were blocked by injecting 60 s-pulses of ethanolamine-HCl (1 M pH 8.5) until complete deactivation. The reference channel was activated and subsequently blocked with ethanolamine. The selected buffers and the immobilized protein levels are reported in Supplementary Table 6. For kinetic and affinity studies with purified antibodies, a CM5 chip was used, and the data was collected at 25 °C at a flow rate of 30 mL/min. The anti-**I**(**Glc**) IgG antibody fraction (at initial concentration tested by absorbance at 280 nm) was diluted in running buffer to final concentrations of 16.6, 33.3, 66.6, and 133.3 nM. Diluted samples were injected over each immobilized antigen according to the method published elsewhere. Kinetic and affinity parameters were obtained using Biacore Evaluation Software 2.0 according to the 1:1 binding model.

### Immunohistochemistry

Lumbar segments of spinal cords from naïve mice, as well as from mice inflicted with EAE induced by the 35–55 peptide of myelin oligodendrocyte glycoprotein (MOG) as described^36^, were paraffin embedded and sectioned at coronal position (4 μ). Deparaffinized and antigen retrieved (citric acid pH 6) sections were pre-incubated in blocking solution containing 20% serum and 0.2% Triton (1 h, RT), and then incubated (72 h, 4 °C) with anti-**I**(**Glc**) IgG antibodies from the representative MS patient serum or with total IgG fraction from the normal blood donor, as well as with rat anti-myelin basic protein antibodies (MBP, Abcam, Cambridge United Kingdom). The second step was performed by labelling with species-specific antibodies (Jackson ImmunoResearch, West Grove, PA, USA), namely, biotinylated anti-human antibody followed by Cy3-conjugated streptavidin, and with a Cy2-conjugated anti-rat antibody. We used 535/40 nm band pass filter for detection of Cy2 and 605/50 nm band pass filter for detection of Cy3. Control slides were incubated with the non-relevant fluorescent secondary anti-IgG antibody. Sections were counter-stained with Hoechst 33258 (Molecular Probes, Eugene, OR, USA) for nuclear staining. Stained sections were examined and photographed by fluorescence microscope (E600, Nikon, Tokyo, Japan), equipped with Plan Fluor objectives connected to CCD camera (DMX1200 F, Nikon). Images were assembled using Adobe Photoshop (Adobe Systems).

## Additional Information

**How to cite this article**: Walvoort, M. T. C. *et al*. Antibodies from multiple sclerosis patients preferentially recognize hyperglucosylated adhesin of non-typeable *Haemophilus influenzae. Sci. Rep.* **6**, 39430; doi: 10.1038/srep39430 (2016).

**Publisher's note:** Springer Nature remains neutral with regard to jurisdictional claims in published maps and institutional affiliations.

## Supplementary Material

Supplementary Information

## Figures and Tables

**Table 1:** Calculated pIC_50_ values for inhibitors of anti-I(Glc).

Inhibitors	MS Sera					NBD sera			
	MS1	MS2	MS3	MS4	MS5	NBD1	NBD2	NBD3	NBD4
I(Glc)	8.65 ± 0.10	8.09 ± 0.56	7.98 ± 0.26	7.57 ± 0.16	7.67 ± 0.28	7.20 ± 0.29	6.92 ± 0.30	6.37 ± 0.52	6.83 ± 0.26
I	<5.0	<5.0	<5.0	7.87 ± 0.24	<5.0	6.86 ± 0.37	7.84 ± 0.42	5.92 ± 0.52	7.09 ± 0.26

Values are reported as calculated pIC_50_ ± the standard error (SEM) in 5 representative MS and 4 NBD sera.

**Table 2:** Calculated pIC_50_ values for inhibitors toward anti-CSF114(N-Glc) IgG antibodies.

	Inhibitor	MS1	MS2	MS3	MS4	MS5	Mean value	95% C.I.*
**1**	**I(Glc)**	9.27 ± 0.07	8.88 ± 0.15	8.42 ± 0.28	9.34 ± 0.19	9.45 ± 0.16	9.07 ± 0.42	8.547–9.597
**2**	**II(Glc)**	8.59 ± 0.04	8.93 ± 0.18	9.94 ± 0.19	9.34 ± 0.10	9.77 ± 0.20	9.31 ± 0.56	8.614–10.01
**3**	**III(Glc)**	8.71 ± 0.11	8.28 ± 0.15	9.34 ± 0.38	9.25 ± 0.12	8.89 ± 0.08	8.89 ± 0.43	8.361–9.427
**4**	**IV(Glc)**	10.14 ± 0.03	8.26 ± 0.08	not tested	not tested	8.56 ± 0.27	8.99 ± 1.01	6.478–11.50
**5**	**V(Glc)**	7.59 ± 0.16	7.21 ± 0.07	6.69 ± 0.14	6.89 ± 0.05	7.56 ± 0.27	7.19 ± 0.40	6.692–7.684
**6**	**I**	<6.0	<6.0	<6.0	<6.0	<6.0	n.a.	n.a.
**7**	**II**	<6.0	<6.0	<6.0	<6.0	<6.0	n.a.	n.a.
**8**	**III**	<6.0	6.80 ± 0.23	<6.0	6.17 ± 1.06	6.44 ± 0.73	6.47 ± 0.32	5.685–7.255
**9**	**IV**	<6.0	<6.0	not tested	not tested	<6.0	n.a.	n.a.
**10**	**V**	<6.0	<6.0	6.41 ± 0.44	<6.0	<6.0	n.a.	n.a.
**11**	**CSF114(N-Glc)**	7.25 ± 0.07	7.17 ± 0.09	8.05 ± 0.26	7.69 ± 0.14	7.66 ± 0.53	7.56 ± 0.36	7.118–8.010

Values are reported as calculated pIC_50_ ± the standard error (SEM) of the N-glucosylated adhesin antigens (**I**(**Glc**)-**V**(**Glc**)) and the corresponding non-glucosylated analogs (**I**-**V**) used as inhibitors of anti-CSF114(N-Glc) antibodies in 5 representative MS sera.

*95% confidence interval (C.I.).
