# Erratum: Antibodies from multiple sclerosis patients preferentially recognize hyperglucosylated adhesin of non-typeable *Haemophilus influenzae*

**DOI:** 10.1038/srep44969

**Published:** 2017-03-22

**Authors:** Marthe T. C. Walvoort, Chiara Testa, Raya Eilam, Rina Aharoni, Francesca Nuti, Giada Rossi, Feliciana Real-Fernandez, Roberta Lanzillo, Vincenzo Brescia Morra, Francesco Lolli, Paolo Rovero, Barbara Imperiali, Anna Maria Papini

Scientific Reports 6: Article number: 39430; 10.1038/srep39430 published online: 12 23 2016; updated: 03 22 2017.

The original version of this Article contained typographical errors in the Abstract.

‘In autoimmune diseases, there have been proposals that exogenous “molecular triggers”, i.e., specific this should be ‘non-self antigens’ accompanying infectious agents, might disrupt control of the adaptive immune system resulting in serious pathologies’.

now reads:

‘In autoimmune diseases, there have been proposals that exogenous “molecular triggers”, i.e., specific ‘non-self antigens’ accompanying infectious agents, might disrupt control of the adaptive immune system resulting in serious pathologies’.

‘The etiology of the multiple sclerosis (MS) remains unclear’.

now reads:

‘The etiology of multiple sclerosis (MS) remains unclear’.

These errors have been corrected in the HTML and PDF versions of this Article.

