# Supplementary material for: Antibodies from multiple sclerosis patients preferentially recognize hyperglucosylated adhesin of non-typeable Haemophilus influenzae
Source: Sci Rep. 2016 Dec 23;6:39430. doi: 10.1038/srep39430 (PMC5180199; doi:10.1038/srep39430)
Supplement: Supplementary Information [file srep39430-s1.pdf]

Supplementary Information

Antibodies from multiple sclerosis patients preferentially recognize hyperglucosylated adhesin of non-typeable *Haemophilus influenzae*

Marthe T C Walvoort[1¥], Chiara Testa[2,3¥], Raya Eilam[4], Rina Aharoni[5], Francesca Nuti[2,6], Giada Rossi[2,3], Feliciana Real-Fernandez[2,3], Roberta Lanzillo[7], Vincenzo Brescia Morra[7], Francesco Lolli[8], Paolo Rovero[2,3], Barbara Imperiali[1]*, Anna Maria Papini[2,6,9]*

[1]Departments of Biology and Chemistry Massachusetts Institute of Technology 77 Massachusetts Ave., Cambridge, MA (USA)

[2]French-Italian Interdepartmental Laboratory of Peptide and Protein Chemistry and Biology (www.peptlab.eu).

[3]Department of Neurosciences, Psychology, Drug Research and Child Health - Section of Pharmaceutical Sciences and Nutraceutics, University of Florence, Via Ugo Schiff 6, 50019 Sesto Fiorentino, Italy.

[4]Department of Veterinary Resources, The Weizmann Institute of Science, Rehovot, Israel. 761001

[5]Department of Immunology, The Weizmann Institute of Science, Rehovot, Israel. 761001

[6]Department of Chemistry "Ugo Schiff", University of Florence, Via della Lastruccia 13, 50019 Sesto Fiorentino, Italy.

[7]Multiple Sclerosis Clinical Care and Research Centre, Department of Neurosciences, Reproductive Sciences and Odontostomatology, Federico II University, Via Sergio Pansini 5, 80131 Naples, Italy

[8]Department of Biomedical, Experimental and Clinical Sciences, University of Florence, Viale Morgagni 50, 50134 Firenze, Italy

[9]PeptLab@UCP and Laboratory of Chemical Biology EA4505, Université Paris-Seine, 5 Mail Gay-Lussac, 95031 Cergy-Pontoise, France

[¥]The authors M.T.C.W. and C.T. contributed equally.

***Corresponding Authors information**:

Barbara Imperiali, PhD
imper@mit.edu

Anna Maria Papini, PhD
annamaria.papini@unifi.it

SUPPLEMENTARY FIGURES

Supplementary Figure 1 Sequence of HMW1ct$_{1205\text{-}1536}$. C-terminal fragment of HMW1A with the 12 putative N-glucosylation sites in brackets and red colored. In blue is the overhang resulting from the vector, and the inserted tryptophan residue.

AHHHHHHV**W**TA^{1205}NSGALTTLAGSTIKGTESVTTSSQSGDIGGTISGGTVEVKATESLTT
QSNSKIKATTGEA**[<u>NVT</u>]$_{1}$**SATGTIGGTISGNTV**[<u>NVT</u>]$_{2}$**ANAGDLTVGNGAEI**[<u>NAT</u>]$_{3}$**EGAA
TLTTSSGKLTTEASSHITSAKGQV**[<u>NLS</u>]$_{4}$**AQDGSVAGSINAA**[<u>NVT</u>]$_{5}$**L**[<u>NTT</u>]$_{6}$**GTLTTVKG
SNI**[<u>NAT</u>]$_{7}$**SGTLVINAKDAELNGAALG**[<u>NHT</u>]$_{8}$**VV**[<u>NAT</u>]$_{9}$**NA**[<u>NGS</u>]$_{10}$**GSVIATTSSRV**[<u>NIT</u>]**
$_{11}$GDLITINGLNIISKNGINTVLLKGVKIDVKYIQPGIASVDEVIEAKRILEKVKDLSDEEREA
LAKLGVSAVRFIEP**[<u>NNT</u>]$_{12}$**ITVDTQNEFATRPLSRIVISEGRACFSNSDGATVCVNIADNG
R^{1536}

Supplementary Figure 2. Data distribution of measured SP-ELISA absorbances. Total anti-**I(Glc)** IgM (**a**) and IgG (**b**) titers and total anti-**I** IgM (**c**) and IgG (**d**) in 126 MS patient sera and 112 NBD sera detected by SP-ELISA. MS patient and NBD sera selected for the detailed analysis are marked as red dots and circles. The IgM antibody absorbance to the glucosylated bacterial adhesin **I(Glc)** (**a**) is higher in MS sera (mean ± SEM 1.001 ± 0.03996) compared to NBD sera (0.779 ± 0.04497; p=0.0003). The IgG antibodies (**b**) discriminated with lower significance (p=0.0141) between MS sera (mean ± SEM 0.9195 ± 0.04674) and NBD sera (0.7645 ± 0.04073). The IgM and IgG antibody levels to the non-glucosylated **I** are similar in MS sera, compared to NBD sera and not statistically significant. These results are in agreement with the proposal that antibodies from NBD sera, that recognize the non-glucosylated adhesin protein **I**, are physiologically present in the sera of most humans due to the prevalence of *H. influenzae*. However, these antibodies are not specific for the N-glucosylated bacterial protein.

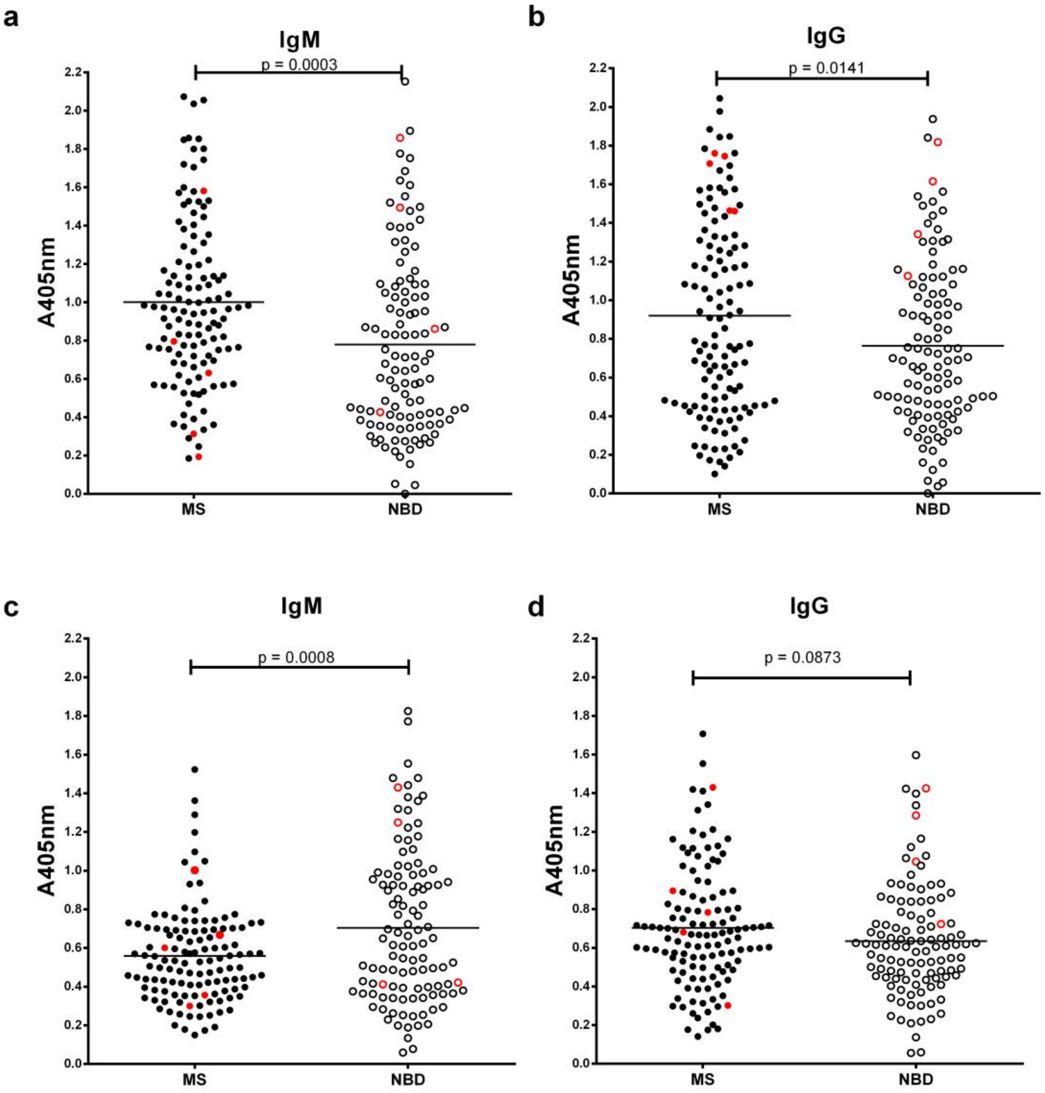

Supplementary Figure 3 Data distribution of measured SP-ELISA absorbances. Total anti-CSF114(N-Glc) IgM (**a**) and IgG (**b**) titers in 126 MS patient sera and 122 NBD sera detected by SP-ELISA. MS patient and NBD sera selected for the detailed analysis are marked as red dots and circles.

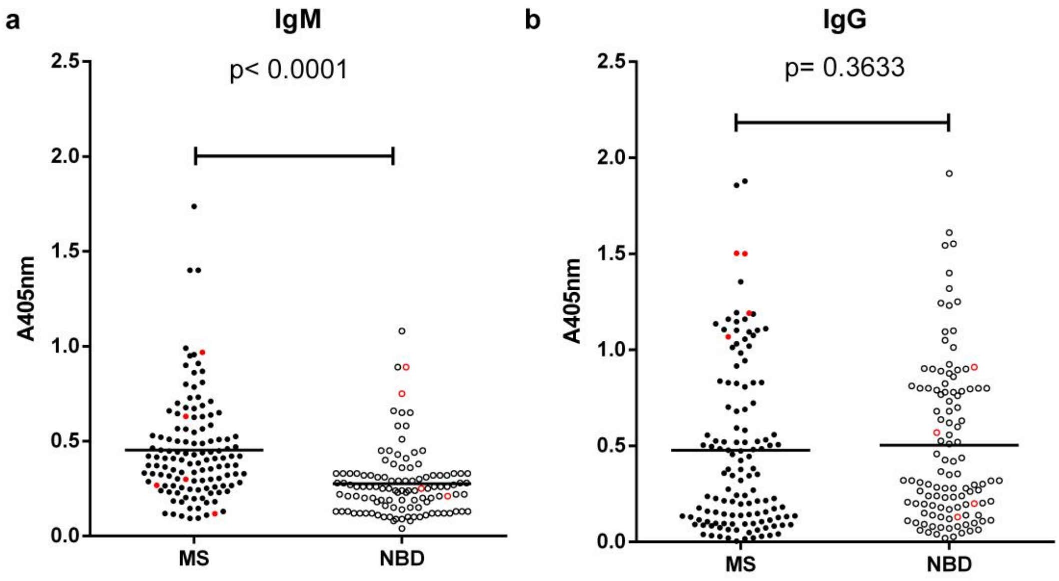

Supplementary Figure 4 Immunoaffinity purification of antibodies from two representative NBD sera. SP-ELISA of antibody fractions obtained from a Sepharose column bearing the immobilized non-glucosylated HMW1ct (**I**). Coated antigen **I** designated as solid bars and coated antigen **I(Glc)** designated as gray bars. Flow through 1 (**FT1**) and eluted fraction 2 (**Elu2**) were collected. Each point is mean±s.d. for n = 3 independent experiments.

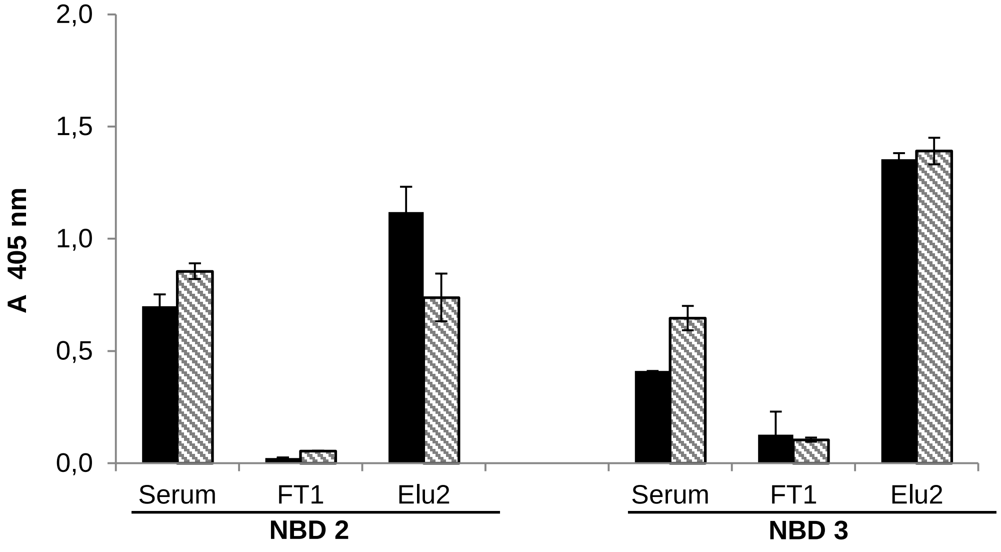

Supplementary Figure 5 SP-ELISA titration of purified antibodies from an MS serum. Titration of purified antibody fractions from a representative MS serum obtained from two sequential Sepharose columns. (**a**) Purified anti-**I** (**Elu2)**. (**b)** Purified anti-**I(Glc) (Elu4)**. Antigen **I** designated as solid bars and antigen **I(Glc)** designated as gray bars.

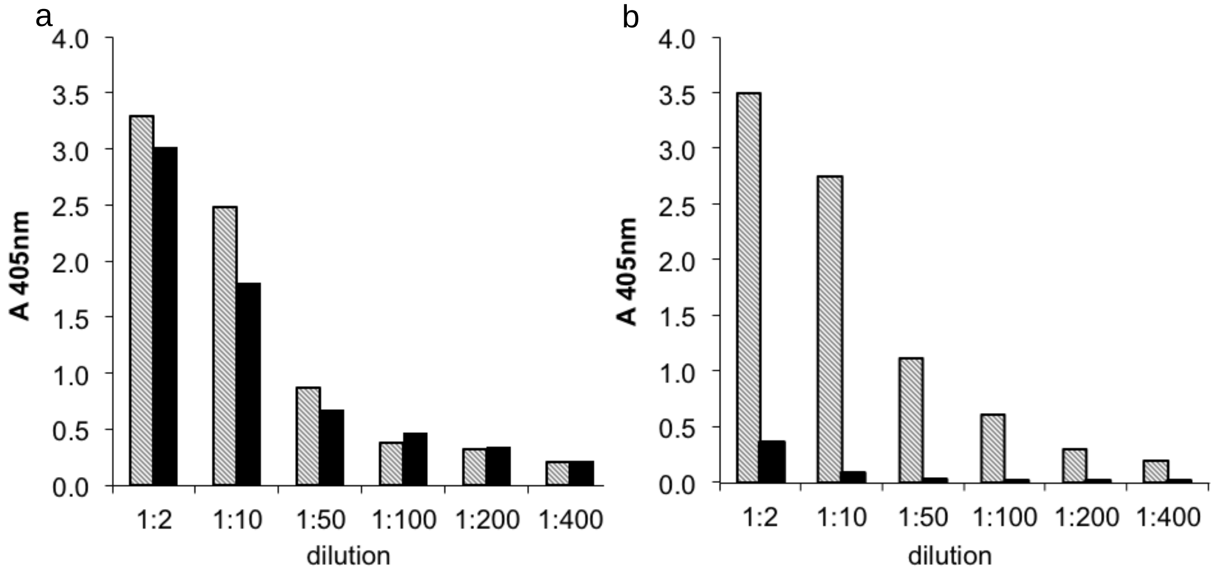

Supplementary Figure 6 Competitive inhibition results of HMW1ct adhesin antigens. Inhibition curves of anti-**I(Glc)** IgG antibodies with the glucosylated HMW1ct adhesin antigens **I(Glc), II(Glc), III(Glc), IV(Glc), V(Glc)**, the corresponding non-glucosylated analogs **I, II, III, IV, V**, and the synthetic antigenic monoglucosylated probe CSF114(N-Glc) The results are shown as percentage of inhibition activity of the representative MS1 serum (ordinate axis) vs. antigen concentrations on a logarithmic scale. The calculated pIC_{50} values are reported in **Supplementary Table 7** as pIC_{50} ± the standard error (SEM).

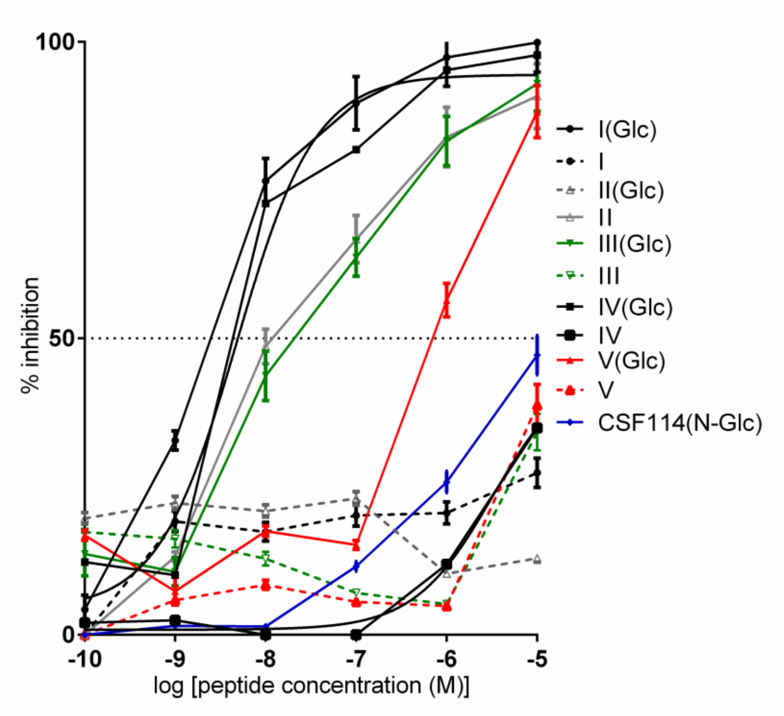

Supplementary Figure 7 Surface Plasmon Resonance experiments of purified anti-I(Glc) antibodies. Sensorgrams displaying the interactions between different concentrations of purified anti-**I(Glc)** antibodies from MS1 serum and both the immobilized antigens **I(Glc)** (**a**) and CSF114(N-Glc) (**b**). Experimental curves are plotted in gray color and fitted curves in black. The calculated affinity constants (K_D) were similar for purified anti-**I(Glc)** antibodies interacting with both immobilized antigens **I(Glc)** [K_D = 2.8 × 10^{-8} M, k_a = (91.8±0.5) × 10^3 $M^{-1}s^{-1}$, k_d = (25.5±0.1) × 10^{-4} s^{-1}, **panel a**] and CSF114(N-Glc) [K_D = 2.6 × 10^{-8} M, k_a = (27.0±0.6) × 10^4 $M^{-1}s^{-1}$, k_d = (69.2±0.9) × 10^{-4} s^{-1}, **panel b**].

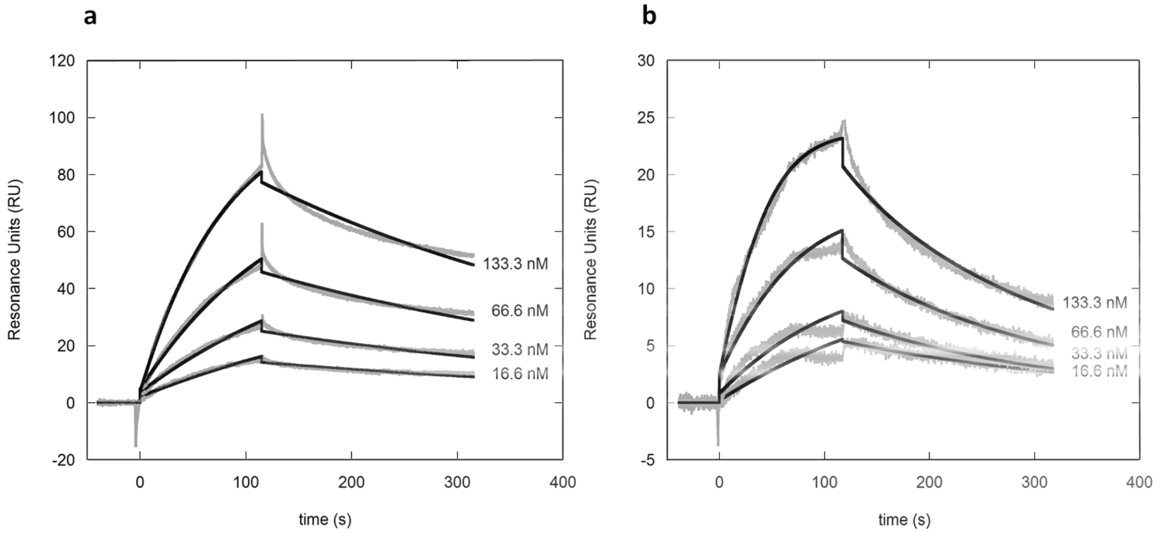

SUPPLEMENTARY TABLES

Supplementary Table 1. Overview of the HMW1ct mutants, the remaining *N*-glycosylation sites, and the glycosylation pattern obtained with the *in vivo* expression protocol.

***N*-glucosylated antigens**	***N*-glycosylation sites[a]**	**Number of *N*-Glc moieties[b]**
**I(Glc)**	1,2,3,4,5,6,7,8,9,10,11,12	7Glc:8Glc:9Glc (1:1:1)
**II(Glc)**	1,2,3,5,6,9	5Glc:6Glc (1:1)
**III(Glc)**	1,2,5,6	4Glc:5Glc (4:1)
**IV(Glc)**	1,2,3,7	2Glc:3Glc:4Glc:5Glc (2:2:2:1)
**V(Glc)**	5,6[c]	2Glc (1)[c]

[a] N-glucosylation sites are reported with the number indicated in **Supplementary Fig. 1**.

[b] Ratio of the N-glucosyl moieties in the adhesin protein antigens was determined by LC-MS.

[c] Glucosylated *in vitro* according the protocol reported in Online Methods.

Supplementary Table 2. MS/MS analysis of **I(Glc)** fragments after tryptic digest. Glucosyated sites are in blue and non-glucosylated sites are in red.

**Peptide**	**Position**	**MW**	**+2**	**+3**	**+4**	**Measured m/z**	**Int**	**Measured m/z**	**Int**
GSNINATSGTLVINAK	168-183	1558.8315	780.42303	520.61778	390.71515	780.4*	5143	520.61	188
GSNINATSGTLVINAK **(site 7)**	168-183	1720.8843	861.44943	574.63538	431.22835	861.41*	5611	574.62	600
VNITGDLITINGLNIISK	217-234	1897.0884	949.55148	633.37008	475.27938	949.52	186	633.36	110
VNITGDLITINGLNIISK **(site 11)**	217-234	2059.1413	1030.5779	687.38771	515.7926				
FIEPNNTITVDTQNEFATRPLSR	293-315	2662.3351	1332.1748	888.45231	666.59105	888.42*	3802	666.58(666.83)	800
FIEPNNTITVDTQNEFATRPLSR **(site 12)**	293-315	2824.3879	1413.2012	942.46991	707.10425	942.45 (942.778)	493	707.09(707.34)	58
DAELNGAALGNHTVVNATNANGSGSVIATTSSR	184-216	3168.5396	1585.2771	1057.1871	793.14218	None			
DAELNGAALGNHTVVNATNANGSGSVIATTSSR	184-216	3330.5924	1666.3035	1111.2047	833.65538	None			
DAELNGAALGNHTVVNATNANGSGSVIATTSSR	184-216	3492.6452	1747.3299	1165.2223	874.16858	1165.52 (1166.19)*	57	874.40(874.90)	33
DAELNGAALGNHTVVNATNANGSGSVIATTSSR **(sites 8, 9 and 10)**	184-216	3654.6980	1828.3563	1219.2399	914.68178	1219.19 (1219.52)*	1212	914.65(914.90	957
GQVNLSAQDGSVAGSINAANVTLNTTGTLTTVK	135-167	3201.6477	1601.8311	1068.2232	801.4192	None			
GQVNLSAQDGSVAGSINAANVTLNTTGTLTTVK	135-167	3363.7005	1682.8575	1122.2408	841.9324	None			
GQVNLSAQDGSVAGSINAANVTLNTTGTLTTVK	135-167	3525.7534	1763.884	1176.2584	882.44563	1176.23 (1176.89)	493	882.43(882.93)	90
GQVNLSAQDGSVAGSINAANVTLNTTGTLTTVK **(sites 4, 5 and 6)**	135-167	3687.8062	1844.9104	1230.276	922.95883	None			
ATTGEANVTSATGTIGGTISGNTVNVTANAGDLT VGNGAEINATEGAATLTTSSGK	66-121	5165.5084	2583.7615	1722.8434	1292.3844	None			
ATTGEANVTSATGTIGGTISGNTVNVTANAGDLT VGNGAEINATEGAATLTTSSGK	66-121	5327.5612	2664.7879	1776.861	1332.8976	None			
ATTGEANVTSATGTIGGTISGNTVNVTANAGDLT VGNGAEINATEGAATLTTSSGK	66-121	5489.6140	2745.8143	1830.8786	1373.4108	1373.69	7 (1374.19)	Unknown where	
ATTGEANVTSATGTIGGTISGNTVNVTANAGDLT VGNGAEINATEGAATLTTSSGK **(sites 1, 2 and 3)**	66-121	5651.6669	2826.8407	1884.8962	1413.924	1413.96	80 (1414.46)		

Supplementary Table 3. Oligonucleotide primers for Quickchange mutagenesis used in this study to remove the consensus sequences for N-glucosylation[a].

**Mutation**	**Sequence (5'→3')**
**Trp intro**	CACCACCATCACGTGTGGACCGCAAACTCAGGC
**Site 1 T→A**	ACGGGCGAAGCCAATGTTGCCAGCGCAAC
**Site 2 T→A**	GTAACACCGTTAATGTCGCGGCAAACGCTGGTGAT
**Site 3 T→A**	CAACGGTGCAGAAATTAATGCTGCCGAAGGTGCAGC
**Site 4 N→Q**	GTCTTGAGCCGACAGCTGGACCTGGCCTTTTGC
**Site 5 and 6 T→A**	CGATCAACGCAGCTAATGTTGCCCTGAATACCGCGGGCACGCTG ACCACGGTC
**Site 7 N→Q**	CCACGGTCAAAGGTAGTAACATTCAGGCCACCTCCGGT
**Site 8 T→A**	CCCTGGGTAACCACGCGGTGGTTAATGCG
**Site 9 T→A**	CGGTGGTTAATGCGGCGAACGCC
**Site 10 S→A**	GACGAACGCCAATGGCGCGGGTTCCGTGATTGCG
**Site 11 N→Q**	TCGCCGGTGATCTGAACACGCGATGACGTGGTCG
**Site 12 N→Q**	CGTGCGCTTTATTGAACCGCAGAATACCATCACGGTTGATA

[a] While only one sequence is displayed, both native and reverse complement sequences are used in the mutagenesis protocol

Supplementary Table 4. Oligonucleotide primers for PCR used in this study.

**Name**	**Sequence (5'→3')**[a]
**Stop forward**	CGTTGGATCCATGGAAAACGAAAATAAACCG
**Stop reverse**	GCTTACTCGAGTCAATTTTCTTTTAGGAACGC

[a] Restriction sites are underlined

Supplementary Table 5. Plasmids.

**Plasmid**	**Relevant features**	**Reference**
**pET45::*His$_6$-hmw1ct(1,2,3,5,6,8,9,10)***	Wild-type HMW1ct	St. Geme et al. 2010
**pET45::*His$_6$-W-hmw1ct(1,2,3,5,6,8,9,10)***	HMW1ct with extra Trp at N-term	This study
**pET45::*His$_6$-W-hmw1ct(1,2,3,5,6,9)***	HMW1ct with 6 sites remaining	This study
**pET45::*His$_6$-W-hmw1ct(1,2,5,6)***	HMW1ct with 4 sites remaining	This study
**pET45::*His$_6$-W-hmw1ct(5,6)***	HMW1ct with 2 sites remaining	This study
**pET45::*His$_6$-W-hmw1ct(1,2,3,7)***	HMW1ct with 4 sites remaining	This study
**pET24::*hmw1C-His$_6$***	HMW1C with a C-terminal His$_6$-tag	St. Geme et al. 2010
**pET24::*hmw1C***	HMW1C without a His$_6$-tag	This study

Supplementary Table 6. Immobilization buffers to maximize the electrostatic interaction with chip surface.

	I	I(Glc)	CSF114	CSF114(N-Glc)
**Immobilization buffer**	5mM CH_3COONa pH 4.5	10mM CH_3COONa pH 4.5	0.5mM CH_3COONa pH 5	0.1mM CH_3COONa pH 5.5
**RU***	8886	7360	2390	1483

*quantity of molecules immobilized in Resonance Unit (RU).

Supplementary Table 7. Competitive inhibition results of HMW1ct adhesin antigens with **anti-I(Glc)** IgG antibodies.

Inhibitors	I(Glc)	I	II(Glc)	II	III(Glc)	III	IV(Glc)	IV	V(Glc)	V	CSF114(Glc)
pIC_{50} ±SEM*	8.65 ±0.10	<0.5	8.10 ±0.20	<0.5	6.01 ±0.16	<0.5	7.70 ±0.26	<0.5	8.32 ±0.24	<0.5	<0.5

*values are reported as pIC_{50} ± the standard error (SEM)
